# The observed alteration in BCL2 expression following lithium treatment is influenced by the choice of normalization method

**DOI:** 10.1038/s41598-018-24546-1

**Published:** 2018-04-23

**Authors:** Damri Odeya, Agam Galila, Toker Lilah

**Affiliations:** 1 Department of Clinical Biochemistry and Pharmacology, Ben-Gurion University of the Negev, Beer-Sheva, Israel; 2 Psychiatry Research Unit, Faculty of Health Sciences, Mental Health Center, Beer-Sheva, Israel; 3 Department of Psychiatry, University of British Columbia, BC, Canada; 4 Michael Smith Laboratories, University of British Columbia, BC, Canada

## Abstract

Upregulation of B-cell CLL/lymphoma (BCL)2 expression following lithium treatment is seemingly well established and has been related to the neuroprotective property of the drug. However, while demonstrated by some (but not all) studies based on low-throughput techniques (e.g. qPCR) this effect is not reflected in high-throughput studies, such as microarrays and RNAseq. This manuscript presents a systematic review of currently available reports of lithium’s effect on BCL2 expression. To our surprise, we found that the majority of the literature does not support the effect of lithium on *BCL*2 transcript or protein levels. Moreover, among the positive reports, several used therapeutically irrelevant lithium doses while others lack statistical power. We also noticed that numerous low-throughput studies normalized the signal using genes/proteins affected by lithium, imposing possible bias. Using wet bench experiments and reanalysis of publicly available microarray data, here we show that the reference gene chosen for normalization critically impacts the outcome of qPCR analyses of lithium’s effect on *BCL2* expression. Our findings suggest that experimental results might be severely affected by the choice of normalizing genes, and emphasize the need to re-evaluate stability of these genes in the context of the specific experimental conditions.

## Introduction

Upregulation of B-cell CLL/lymphoma (*BCL*)*2* transcript following chronic lithium treatment is considered to be well established^1^. Chen *et al*. and Chen and Chuang^2,3^ were the first to report lithium-induced increased gene and protein expression of *BCL2* following lithium treatment. Due to the anti-apoptotic effect of BCL2^4^ this upregulation has been interpreted as a possible mechanism underlying at least in part the neuroprotective property of the drug (reviewed in^5^).

However, BCL2’s function is not restricted to prevention of apoptosis. In the endoplasmic reticulum (ER) BCL2 is a potent inhibitor of autophagy^6^, a vital cellular process shown to be augmented by lithium^7^. Indeed, we have found that lithium-treated mice exhibit changes in protein levels of autophagy markers (upregulation of Beclin-1 and downregulation of p62) indicative of upregulation of this process^8^. Thus, the well accepted lithium-induced upregulation of BCL2, while corroborating other pro cell-survival effects of the drug, contradicts its pro-autophagy effect. Moreover, the lack of evidence for increased risk of cancer or poorer malignancy prognosis among lithium-treated subjects^9,10^ is counterintuitive with the anti-apoptotic consequence of increased BCL2 expression^4,11^ and with the positive correlation between malignancy and BCL2 expression^12,13^.

We have recently performed a real-time PCR quantification of the expression levels of several genes previously reported to be affected by lithium in hippocampi of chronically lithium-treated mice and of inositol monophosphotase-1 (IMPase-1, a lithium-inhibitable enzyme at therapeutically-relevant concentrations, encoded by *IMPA1*) homozygote knockout (KO) mice^14^. *BCL2* was one of the genes examined in this study. Guided by a recent study demonstrating that stability of commonly used normalizing genes (e.g. *GAPDH*, *ACTB*) is affected by mood-stabilizer treatment, we used *MAPK6* to normalize our data. *MAPK6* was recommended as a brain-reference gene^15^, and we validated its stability using unpublished data from our microarrays study of similar lithium treatment conditions (i.e. regimen, tissue and mouse strain)^16^. Surprisingly, we observed significantly decreased rather than increased *BCL2* mRNA levels, both following lithium treatment and in *IMPA1* KO mice. In the search for a plausible explanation for our finding we carefully reviewed studies in which lithium’s effect on *BCL2* transcript and protein levels was assessed. As summarized in Table 1, a laborious screening of previous studies revealed that upregulation of BCL2 by lithium is far from being robust. Rather, *BCL2* upregulation was not observed in any of the microarrays studies we screened^17–22^. We have also noticed that in some of the studies supporting lithium-induced upregulation of *BCL2* the reference genes used for normalization have previously been reported to be affected by lithium (e.g. *ACTB*, *GAPDH*, *TUBB*, Tables 1 and 2).

**Table 1:** Previous studies assessing the effect of lithium on BCL2 levels.

Specie	Tissue	Dose	Regime	N	Assay	Norm	Effect size	Reference
Rat	FC	4 meq/kg/day **4 meq/kg/day**	9 days **4 weeks**	10 **10**	WB **WB**	NA **NA**	NS +2.1*	^3^
Rat	CGC	0.5–5 mM 0.5 mM 1–5 mM	7 days 7 days 7 days	3 3 3	NB WB WB	NA NA NA	NS NS+ 1.6*/+2.5*	^2^
Rat	DG	4 meq/kg/day **4 meq/kg/day**	2 days **4weeks**	4 **4**	IHC **IHC**	NA **NA**	NS +1.4*	^30^
**Rat**	**RGC**	**0.2–5 mM**	**5 days**	**1**	**PCR**	***Gapdh***	**increase**	^55^
**Rat**	**DG**	**4 meq/kg/day**	**4 weeks**	**5**	**IHC**	**NA**	+1.25**	^31^
**Rat**	**RGC**	**30 mg/kg/day**	**7 days**	**NA**	**IHC**	**NA**	**increase**	^75^
Rat	DG CA1	1 meq/kg/day 1 meq/kg/day	2 weeks 2 weeks	9 9	ELISA ELISA	NA NA	NS NS	^76^
Rat	**DG** **CA1**	**1 meq/kg/day** **1 meq/kg/day**	**4 weeks** **4 weeks**	**6** **6**	**ELISA** **ELISA**	**NA** **NA**	+1.33*** +1.15*	^77^
Rat	FC	50 mg/kg/day	21 days	20	MA	NA	NS	^20^
Rat	PNC PAC PNAC PNC **PAC** PNAC	1 mM 1 mM 1 mM 1 mM **1 mM** 1 mM	7 days 7 days 7 days 7 days **7 days** 7 days	7 7 7 7 **7** 7	qPCR qPCR qPCR ELISA **ELISA** ELISA	*Gapdh* *Gapdh* *Gapdh* NA **NA** NA	NS NS NS NS +2.75* NS	^56^
Rat	HP	20–63 mg/kg/day	5 days	4	qPCR	*Rpl24*	NS	^78^
**Rat**	**FC** **HP**	**47 mg/kg/day** **47 mg/kg/day**	**13 days** **13 days**	**10**	**WB** **WB**	**Actb** **Actb**	+1.4*** +1.3***	^58^
Mouse	Brain	4 g/kg chow	2 weeks	10	MA	NA	NS	^17^
Mouse	Brain	8 mmol/kg/day 8 mmol/kg/day	7 days 7 days	6	qPCR MA	*Gapdh* NA	NS NS	^18^
Mouse	HSPC	5 mM	7 days	3	MA	NA	NS	^21^
Mouse	FC **HC**	2–4 g/kg chow **2–4 g/kg chow**	2 weeks **2 weeks**	13	qPCR **qPCR**	*Mapk6* ***Mapk6***	NS −1.2*	^14^
Mouse	MSC MSC	2.5 mM 2.5 mM	24 h 24 h	4 4–6	MA qPCR	NA *PPIA*	NS NS	^22^
**Chick**	**NM**	**1.5–3 mM/kg**	**17 days**	**6**	**IHC**	**NA**	+1.5*	^35^
Human (BP)	Blood	>300 mg >300 mg	2–8 weeks 2–8 weeks	10	MA qPCR	NA NA	NS NS	^59^
Human (Manic)	Serum	900–1200 mg/day@	~month	20	ELISA	NA	NS	^79^
Cell line	hNT SVG SVG	0.75–2 mM 0.75–2 mM 0.75–2 mM	7 days 7 days 7 days	24	qPCR qPCR WB	18s rRNA 18s rRNA NA	NS NS NS	^67^
**Cell line**	**PC12**	**2 mM**	**7 days**	**3**	**WB**	**NA**	+1.2*	^28^
**Cell line**	**SH-SY5Y**	**1 mM**	**7 days**	**5**	**WB**	**β-tubulin**	1.65*	^57^
Cell line	SK-N-AS	1.5 mM	33 days	5	MA	NA	NS	^19^
Cell line	SH-SY5Y	1 mM 1–2 mM **3–5 mM**	6 h/72 h 48 h **48 h**	4 4 **4**	MA qPCR **qPCR**	NA NA **NA**	NS NS+ +1.6*/+2.2**	^29^
Cell line	SH-SY5Y	1 mM	6 h	9	qPCR	18s rRNA	NS	^80^
**Cell line**	**SH-SY5Y**	**2 mM** **2 mM**	**12 h** **12 h**	**5** **5**	**qPCR** **WB**	***Gapdh*** **β-Actin**	+3.5* +1.7*	^27^
*C*. *elegans*		10 mM	life-time	6	MA	NA	NS	^81^

@ - combined with additional medications; *p < 0.05, **p < 0.01, ***p < 0.001, all significant results are indicated in bold. Norm – normalizing gene/protein; WB – Western blotting; MA – Microarray; NB – Northern blotting; IHC – immunohistochemistry; CGC – cerebellar granular cells; HSPC - hematopoietic stem/progenitor cell; MSC – mesenchymal stem cells; PAC – primary astrocyte culture; PNC – primary neuronal culture; PNAC - primary mixed neuro-astrocyte culture RGC – retinal ganglion cells; CA1 – hippocampal area CA1; DG – dentate gyrus; FC – frontal cortex; HP – hippocampus; NM – nucleus magnocellularis (avian cochlear nucleus); NA – not applicable/not available; NS – non-significant.

**Table 2:** Differential mRNA and protein expression of genes/proteins commonly used to normalize mRNA and protein levels found in microarrays and proteomics studies.

Specimen	Gene/protein	Name	Relative change	Reference
Hippocampus BD	protein	Actb, Tubb	CA1: + 1.19, −1.14 CA2/3: −1.3, −1.19 DG: −1.225, −1.11	^44^
Hippocampus SCZ	protein	Actb, Tubb	CA1: + 1.1, −1.08 CA2: NS, −1.09 DG: −1.176, −1.1	^44^
ACC MDD	protein	Tuba	+1.81	^42^
ACC BD	protein	Tubb	−1.35	^42^
DPC BD	protein	Actb, Tubb	−1.46, −1.12	^43^
DPC SCZ	protein	Tuba, Tubb	+1.3, +1.42	^43^
Lithium-treated mice, brain	mRNA	Actb	+7.479	^18^
Lithium-treated rats, PFC synaptosomes	mRNA	Tubb, Gapdh	−0.48, downregulated*	^48^
Lithium-treated rats, PFC	protein	Gapdh	−1.27	^50^
Lithium-treated rats, IMCD	protein	Actb	+1.7	^49^
Lithium-treated mice, brain	mRNA	Tuba4	+1.3	^17^
Lithium-treated rats, brain	mRNA	Tuba	+2.2	^46^
Ischemia, mouse brain synaptosomes	protein	Actb, Gapdh	+1.6, +2.4,	^51^
Lithium-treated, mice, FC	Protein	Tubb3, Tubb4, Tubb5	+1.07, +1.05, +1.04	Toker *et al*. (under preparation)
Lithium-treated mice, FC	mRNA	Actb, Tuba8, Tubb2a, Tubb2c, Tuba1a, Tuba1b, Tuba4a	−1.11, +1.67, 1.24, 1.17, 1.12, 1.06, 1.2	^16^ (unpublished data, available upon request)

ACC- anterior cingulate cortex; DG – dentate gyrus; DPC – dorsolateral prefrontal cortex; IMCD – inner modullary collecting ducts; PFC – prefrontal cortex; BD – bipolar disorder; SCZ – schizophrenia; Actb – β-actin; Tuba – α-tubulin; Tubb – β-tubulin; Gapdh – glyceraldehyde phosphate dehydrogenase.

* - fold of change not reported.

We therefore hypothesized that the discrepancy between microarrays and qPCR studies may, in part, result from the need to normalize qPCR expression using a chosen reference gene. To test our hypothesis we assessed the effect of different normalizing genes on lithium-induced *BCL2* expression measured using qPCR. To further validate our results we also assessed the impact of normalizing genes on the expression of another gene known to be affected by lithium - *Myristoylated Alanine Rich Protein Kinase C Substrate* (*MARCKS)*^17,23–25^.

## Results

### The effect of lithium on BCL2 and MARCKS expression levels when normalized to ACTB

**Figure 1:**
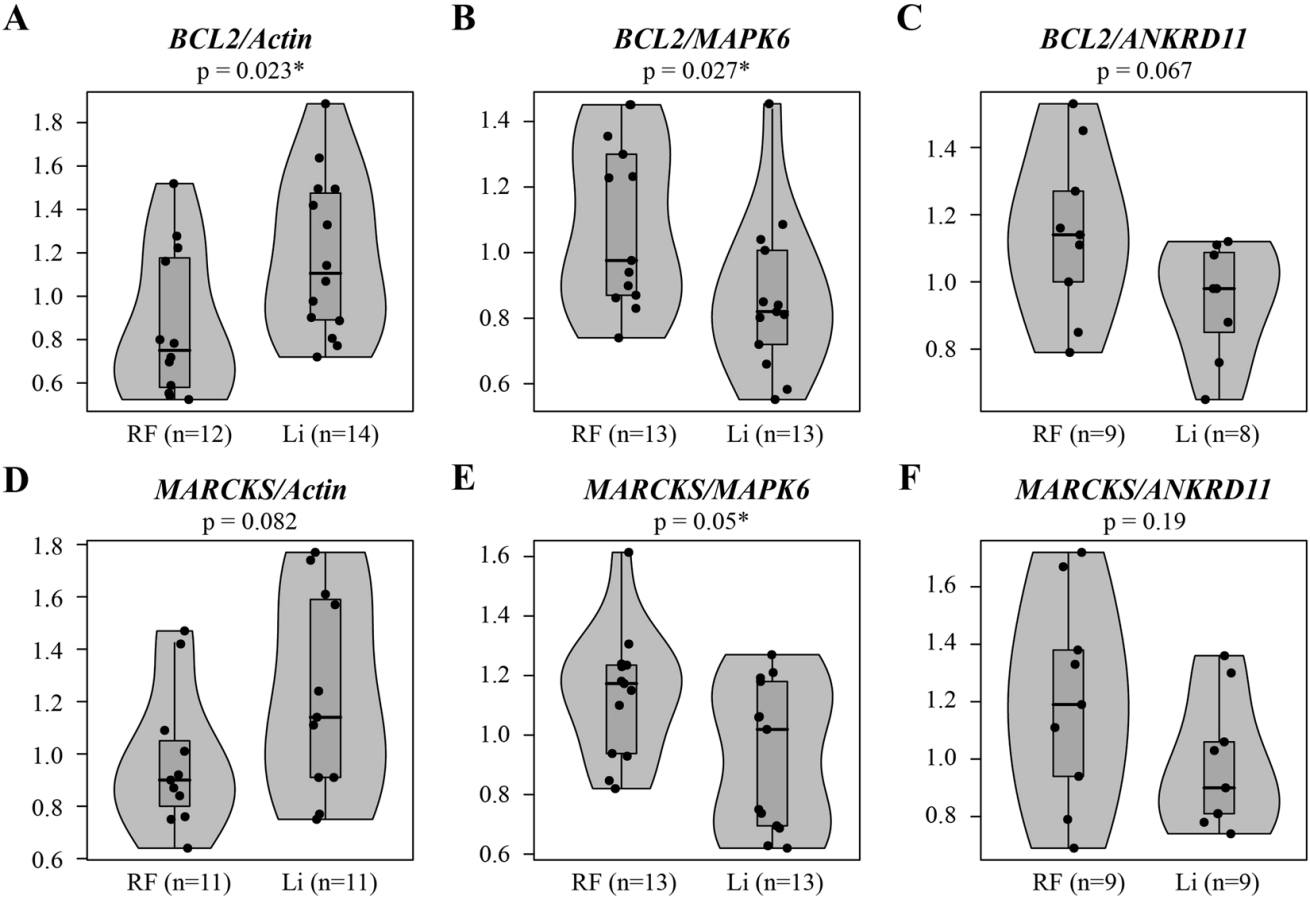
The direction of the observed effect of lithium treatment on gene expression depends on the normalizing gene. Violin plots of hippocampal *BCL2* (upper lane) and *MARCKS* (lower lane) mRNA levels of control mice and of chronic lithium-treated mice, normalized to different normalizing genes. When normalized to *β-Actin* (**A**,**D**), both *BCL2* and *MARCKS* appear to be upregulated by lithium treatment. However, following normalization to *MAPK6* (**B**,**E**), both transcripts appear to be downregulated by lithium treatment. Normalization to *ANKRD11* (**C**,**F**) supports the results obtained following normalization to *MAPK6*. RF – regular food; Li – lithium treatment. Each point represents an individual normalized value, Boxplots show the median and the interquartile range. p-values were calculated using two-sided Wilcoxon rank sum test. *p < 0.05.

*BCL2* levels normalized to *β-Actin* were higher in the lithium treatment group than in the regular food group [regular food *vs*. lithium treatment, median (interquartile): 0.75 (0.58, 1.17) *vs*. 1.01 (0.89, 1.48), location shift (95%CI): 0.28 (0.05 to 0.63), p = 0.023, Fig. 1A]. No significant difference was found in normalized *MARCKS* levels [regular food *vs*. lithium treatment: 0.9 (0.80, 1.05) *vs*. 1.14 (0.91, 1.59), location shift (95%CI): 0.23 (−0.01 to 0.65), p = 0.081, Fig. 1D].

### The effect of lithium on BCL2 and MARCKS expression levels when normalized to MAPK6

*BCL2* levels normalized to *MAPK6* were lower in the lithium treatment group than in the regular food group [regular food *vs*. lithium treatment: 0.98 (0.87, 1.30) *vs*. 0.82 (0.72, 1.00), location shift (95%CI): −0.21 (−0.44 to −0.02), p = 0.027, Fig. 1B]. Similarly, *MARCKS* levels normalized to *MAPK6* were lower in the lithium treatment group than in the regular food group [regular food *vs*. lithium treatment: 1.17 (0.93, 1.24) *vs*. 1.01 (0.69, 1.18), location shift (95%CI): −0.18 (−0.44 to 0.007), p = 0.050, Fig. 1E].

### Estimation of the expression stability of genes in mouse hippocampus

**Figure 2:**
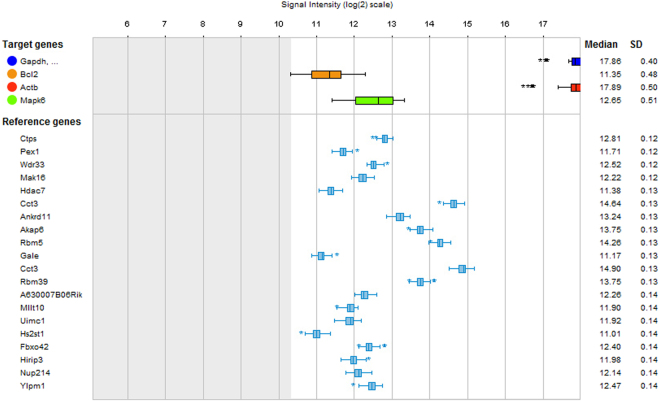
Expression stability of *β-actin*, *GAPDH* and *MAPK6* in hippocampus of WT-untreated mice is similar to that of *BCL2*. A snapshot obtained using the RefGenes tool of the Genevestigator software. Based on 80 microarrays of hippocampal samples of WT untreated mice the signal variability of the commonly used normalizing genes is similar to that of *BCL2*, as can be noticed from the SD of the log_2_ transformed signals. The box-plots represent the ranges of the log_2_-transformed signals of the suggested reference genes (light blue), the commonly used reference genes (*GAPDH*, *β-actin* and *MAPK6* - dark blue, red and green, respectively) and of *BCL2* (orange). Asterisks indicate outliers’ expression values.

We utilized the RefGenes tool of the Genevestigator software that ranks genes based on the variance in their expression in a chosen set of samples from microarrays database. We searched for the most stable genes in the hippocampus of wildtype (WT) mice and estimated the stability of *BCL2* and three reference genes: *ACTB*, *GAPDH* and *MAPK6*. As reflected by the standard deviation (SD) of the log_2_ transformed signals (Fig. 2) the expression stability of *β-actin*, *GAPDH* and *MAPK6* in WT untreated mice is similar to that of *BCL2* (SD: 0.4, 0.51, 0.5 and 0.48, respectively).

### The effect of lithium on BCL2 and MARCKS expression levels when normalized to ANKRD11

We used the list of the most stable reference genes in mouse hippocampus obtained from the RefGenes tool (Fig. 2) to select an additional normalizing gene for real-time PCR analysis of *BCL2* and *MARCKS*. We further evaluated the stability of the top suggested reference genes in our (unpublished) microarrays data of Toker *et al*.^16^. Among these genes *ANKRD11* was the most stable gene that was not significantly affected by lithium treatment or *IMPA1* KO in our microarray study (lithium treated WT mice from the SMIT1 colony: p > 0.8; lithium treated WT mice from the IMPA1 colony: p > 0.14). In agreement with the results obtained when normalized to *MAPK6* (Fig. 1B,E), *BCL2* and *MARCKS* levels normalized to *ANKRD11* were lower in the lithium treatment group than is the regular food group: *BCL2* - regular food *vs*. lithium treatment: 1.14 (1.00, 1.27) *vs*. 0.98 (0.85, 1.087), location shift (95%CI): −0.17 (−0.45 to 0.01), p = 0.067, Fig. 1C; *MARCKS* - regular food *vs*. lithium treatment: 1.19 (0.94, 1.38) *vs*. 0.9 (0.81, 1.06), location shift (95%CI): −27 (−0.57 to 0.11), p = 0.185, Fig. 1F.

### Lithium’s effect on the expression of BCL2 and MARCKS based on publicly available microarrays datasets

Genes on microarray platforms are often represented by several probesets, targeting different parts of the gene (Table 3). None of the probesets representing the *BCL2* gene was significantly affected by lithium treatment in the two analyzed datasets (non-adjusted p-value > 0.1, adjusted p-value > 0.5). The expression of *MARCKS* was significantly downregulated by lithium in the GSE35291 dataset (adjusted p-value = 5.5*10^−3^). None of the probesets corresponding to *MARCKS* in the GSE66277 dataset showed a significant effect of lithium after controlling for FDR (adjusted p-value > 0.2). However, in the hippocampal samples, in two out of the six probesets, *MARCKS* levels were downregulated (non-adjusted p-value < 0.025). Table 3 summarizes the results of differential analyses for all *BCL2* and *MARCKS* probesets in the two datasets.

**Table 3:** Differential expression analysis of *BCL2* and *MARCKS* probesets in two publicly available datasets.

Dataset	Tissue	Probeset	Gene	logFC	AveExpr	t	p-value	Adjusted p-value	B
GSE66277 (Rat)	Cortex	1387611_at	*BCL2*	0.085	4.896	1.005	0.322	0.587	−5.700
		1370948_a_at	*MARCKS*	−0.129	9.963	−1.298	0.203	0.464	−5.381
		1373432_at	*MARCKS*	0.085	11.098	1.147	0.260	0.528	−5.556
		1388157_at	*MARCKS*	−0.096	7.457	−0.771	0.446	0.692	−5.901
		1375523_at	*MARCKS*	0.044	10.668	0.484	0.632	0.819	−6.077
		1370949_at	*MARCKS*	0.009	9.680	0.080	0.937	0.974	−6.189
	Hippocampus	1387611_at	*BCL2*	0.059	4.834	0.704	0.486	0.795	−5.786
		1375523_at	*MARCKS*	−0.185	10.655	−2.575	0.015	0.198	−3.113
		1370948_a_at	*MARCKS*	−0.214	9.685	−2.364	0.024	0.243	−3.532
		1370949_at	*MARCKS*	−0.162	9.489	−1.694	0.100	0.435	−4.692
		1373432_at	*MARCKS*	−0.110	10.751	−1.661	0.106	0.445	−4.741
		1388157_at	*MARCKS*	−0.122	7.276	−1.124	0.269	0.640	−5.424
	Striatum	1387611_at	*BCL2*	−0.020	5.103	−0.234	0.817	0.950	−5.805
		1373432_at	*MARCKS*	0.040	11.358	0.663	0.512	0.831	−5.625
		1370949_at	*MARCKS*	0.046	9.977	0.506	0.616	0.880	−5.710
		1375523_at	*MARCKS*	−0.031	11.138	−0.449	0.656	0.897	−5.735
		1388157_at	*MARCKS*	−0.030	7.722	−0.252	0.803	0.944	−5.801
		1370948_a_at	*MARCKS*	0.000	10.227	−0.003	0.998	1.000	−5.830
GSE35291 (Mouse)	Hematopoetic stem/progenitor cells (HSPC)	ILMN_2597567	*BCL2*	0.179	7.024	1.613	0.136	0.569	−5.554
		ILMN_2682162	*BCL2*	0.167	7.795	1.231	0.245	0.717	−6.045
		ILMN_1249021	*BCL2*	0.189	8.078	1.093	0.298	0.764	−6.199
		ILMN_1215796	*BCL2*	0.116	7.092	0.987	0.345	0.796	−6.307
		ILMN_1249366	*BCL2*	0.163	7.743	0.757	0.465	0.863	−6.509
		ILMN_2706514	*BCL2*	−0.076	7.445	−0.480	0.641	0.925	−6.687
		ILMN_1243345	*BCL2*	−0.017	6.735	−0.266	0.795	0.964	−6.772
		ILMN_2597272	*BCL2*	−0.017	6.594	−0.210	0.838	0.972	−6.786
		**ILMN_1256142**	***MARCKS***	**−0.896**	**8.986**	**−6.070**	**8.79E-05**	**5.50E-03**	**1.706**

## Discussion

In this study we show that conclusions of experiments assessing the effect of lithium treatment on *BCL2* expression can noticeably be affected by different experimental set-ups. In qPCR experiments normalization with *ACTB* suggested significant upregulation of the gene, supporting the common notion of lithium’s treatment effect on this gene. Contrarily, normalization of the same samples with either *MAPK6* or *ANKRD11* suggested significant downregulation, contradicting the common notion. Alternatively, our analysis of publicly available microarray data from lithium treated rodents showed no apparent effect of the drug on *BCL2* expression, in line with previous microarray studies of lithium’s effects^17–22^.

Our findings are surprising in light of the common notion that lithium treatment induces upregulation of *BCL2* transcript and protein levels, interpreted to contribute to the neuroprotective effect of the drug. However, thorough examination of reported data reveals that the effect is not as robust as generally perceived. Thirty one out of 49 (63%) published experimental set-ups found no significant effect and only 15 supported significant upregulation of *BCL2* transcript/protein. In four out of the latter 15 experimental set-ups the raw data was normalized to genes/proteins previously shown to be affected by lithium (Tables 1, 2). The remaining three experimental set-ups (out of the 49 ones) either did not provide information regarding the significance of the finding or reported downregulation of the gene.

There are several possible reasons for the discrepancies among the different studies, emphasizing the uncertainty in our knowledge of the truth regarding lithium’s effect on *BCL2* transcript and/or protein levels.

### Lithium treatment regime

The first to report lithium-induced *BCL2* upregulation in rat brain were Chen and colleagues^3^. The effect was found following four weeks but not nine days of treatment with a high lithium dose − 4 meq/kg/day. The authors reported that this treatment resulted in therapeutically-relevant lithium blood levels. However, an earlier study using this dose^26^ reported dissimilar pharmacokinetics of the drug in plasma *vs*. brain during a prolonged treatment period (21 days); in plasma, after two weeks of treatment, the authors found a gradual decrease in the drug’s concentration. In contrast, in the brain, lithium levels increased continuously during the whole treatment period. This suggests that brain lithium levels in the Chen *et al*.’s study^3^ might have been higher than the therapeutic range of the drug. Lithium doses exceeding the range used for therapeutic purposes were used in several additional studies supporting lithium-induced upregulation of *BCL2* transcript/protein^27–31^.

### Possible misinterpretation of the findings

The possibility that the observed increase in BCL2 protein merely reflects lithium-induced cell proliferation^32,33^ cannot be ruled out. Based on their immunohistochemistry results, Chen *et al*.^3^ report “increased number of BCL2 positive cells” rather than increased BCL2 intensity. Interestingly, a similar treatment protocol was later used to study the effect of lithium on long-term potentiation (LTP) and neurogenesis^30,31^. The authors report 50% increase in newborn cells in the dentate gyrus, accompanied by ~40% increase in BCL2 protein levels in the same region. While a causative effect of BCL2 upregulation on cell proliferation/neurogenesis cannot be ruled out, Bernier and Parent^34^ have previously shown that BCL2 is a marker of immature neurons, which are likely to dominate in the dentate gyrus, where neurogenesis occurs.

An additional study frequently cited in support of lithium-induced upregulation of BCL2 examined the drug’s potency to protect chick cochlear nucleus neurons from deafferentiation-induced apoptosis^35^. In this model cessation of protein synthesis and upregulation of *BCL2* transcript precede neuronal death^36,37^. As reported by Bush and Hyson^35^ chronic lithium treatment reduced cellular death and increased BCL2 protein levels in the deafferentiated cochlear nucleus. The authors speculated that increased BCL2 levels may have resulted from lithium-induced activation of specific transcription factors (TFs). Although generally plausible, regulation of TFs activity is unlikely to take place in a scenario of protein synthesis arrest. Rather, a more likely interpretation (in light of the increased *BCL2* transcript in these cells prior treatment) is cellular recovery from cessation of protein synthesis as a result of lithium treatment. Indeed, it has been demonstrated that inhibition of protein synthesis in the deafferentiated cochlear nucleus results from phosphorylation of elongation factor 2 (eEF2)^38,39^ that can be reversed by lithium treatment^40^. It is thus conceivable that the observed lithium-induced upregulation of BCL2 in Bush and Hyson’s study^35^ does not reflect a specific upregulation of the BCL2 protein, but, rather, a general increase in protein synthesis.

### The choice of the normalizing gene/protein

Among common normalizing genes *MAPK6* has been suggested to be the most appropriate for brain qPCR analyses^15,41^. Other commonly used reference genes, for example *GAPDH*, *ACTB*, *TUBA* and *TUBB* have been repeatedly reported as altered in bipolar patients^42–44^, following lithium treatment^17,18,45–50^ or oxidative stress^51^. In addition, the tubulin proteins are involved in autophagy^52,53^, a cellular pathway known to be affected by lithium^7^. In our own microarrays and proteomics analyses (Table 2) mRNA and protein levels of GAPDH, β-Actin and α/β-Tubulin were affected in lithium-treated mice. Stability of different normalizing genes following acute and chronic treatment with mood stabilizers was recently evaluated by Powel *et al*.^54^. The authors show that neither of the genes shows stable expression following treatment with these drugs. Specifically, *GAPDH* and *ACTB* were among the least stable genes following chronic lithium treatment. Despite all of the above, in a substantial proportion of the reviewed studies data was normalized using genes/proteins shown to be affected by lithium treatment^18,27,55–58^.

### Biased choice of authors which of the results to emphasize

A cautious analysis of Lowthert *et al*.’s report^59^ suggests that at least in some cases the authors only concentrate on favorable findings. Studying lithium-responsive and non-responsive bipolar patients the authors report upregulation of *BCL2* in lithium-responders and downregulation in non-responders one month following treatment. In essence, they used microarrays to study peripheral blood gene expression in the patients over a two-month period. Blood samples were taken at baseline and every two weeks. Similarly to other microarrays studies of lithium’s effect^17–22^ the microarray data did not support *BCL2* expression changes in any of the time points. Nevertheless, the authors performed qPCR analysis of the *BCL2* gene family. No change was found in *BCL2* expression neither in lithium-responders nor in non-responders as compared to untreated healthy subjects. The finding reported by the authors is based on an increased ratio of *BCL2* transcript between lithium-responders and non-responders found at a single time point - one month after treatment initiation. This is regardless of the data showing that at the remainder of the time points (4 out of 5) *BCL2* expression was similar or lower in the responders, both as compared to non-responders or to healthy subjects^59^.

### Impact of normalizing genes/proteins in biological psychiatry

The discrepancy between microarray and qPCR findings and the contradicting results obtained by normalizing the same data using different ‘housekeeping’ genes raise the question regarding the impact of normalising genes on qPCR analyses in biological psychiatry. Several studies assessed the issue of “compatible” reference genes for qPCR, in various tissues, in general^15,60,61^, and for brain, in particular^41,62^. The take-home message of these and other tissue-specific studies is that there are no common reference genes. Rather, reference genes should be chosen *de-novo* for each species/tissue/condition studied. The present data obtained for WT untreated mouse hippocampus based on the RefGenes tool point out that the expression of reference genes commonly used in qPCR analyses are as unstable as the genes of interest. As discussed by Hugget *et al*.^63^ inter-group variability in normalizing gene expression is acceptable when the group effect on the expression of the gene of interest is substantially larger. In the field of biological psychiatry the effect sizes rarely exceed 1.5 fold^64^. According to the RefGenes tool, the lowest standard deviation (SD) of log_2_-transformed signals in mouse hippocampus is 0.12, and the SD of the commonly used housekeeping genes is ~0.45 (relative fold change of 1.18 and 1.8, respectively). Thus, changes in the expression level of genes of interest may be obscured by the variation in the expression of the normalizing gene. Moreover, our results suggest that the biological effect of the studied condition on the reference gene chosen may be larger than the effect on the gene of interest, resulting in significant but erroneous results. Similar findings in different tissues were reported by others^65,66^.

Stability of reference genes in control animals (namely, the SD of the genes’ expression in WT untreated animals) provides information regarding the within-group variability. Though ideally one should estimate the stability of the reference genes in both the control and the treatment groups, it is unlikely that microarrays data from a sufficient number of similar treatment samples is obtainable. Nevertheless, it is reasonable to assume that the stability of given reference genes in treated samples are similar to, or lower than that in control samples. Differences in the response to treatment of individual subjects/samples as well as potential differences in the actual treatment dose (e.g. injection volume deviations, variation in drug-containing food consumption) are additional sources of variation in gene expression.

To sum up, here we show that qPCR quantification of the effect of two weeks lithium treatment on *BCL2* and *MARCKS* expression strongly depends on the normalizing gene chosen. Specifically, both genes exhibit downregulation when normalized to *MAPK6* or *ANKRD11*, but upregulation when normalized to *β-Actin*. Inverse results obtained using different normalizing genes have previously been reported by others^62^. Our literature search as well as analysis of two publicly available datasets of lithium treatment revealed inconsistency among previous studies reporting an increase in BCL2 levels following lithium treatment. Moreover, the majority of studies supporting this increase either: (a) used lithium doses exceeding therapeutically-relevant ones; (b) normalized the signals to genes/proteins shown to be affected by lithium; or (c) used a small sample size. We also note that the increase in BCL2 expression was not supported by any of the numerous microarrays studies.

We therefore suggest that reports regarding lithium’s effect on BCL2 expression be considered with caution. While it cannot be ruled out that under some conditions lithium treatment increases BCL2 expression, this effect may not be a general and reproducible one^57,67^. According to our literature search this effect was primarily observed in rats at a four-week time point using treatment regimen that potentially results in higher than the therapeutically-relevant levels. Due to the complex nature of lithium’s mode of action and the diversity of BCL2’s functions, an effort should be invested in the experimental design, report, and interpretation of future studies. For example, rather than measuring *BCL2* mRNA or protein levels in whole cell lysates, isolated subcellular organelles such as mitochondria, endoplasmic reticulum, nuclei, or cytosol might provide more consistent results. Furthermore, as lithium induces cell proliferation, and as BCL2 is also expressed in newly-born cells, BCL2 levels might better be normalized to cell count whenever possible. When normalization to specific genes or proteins is carried out, verification that they are not affected by the condition studied is mandatory. In addition, providing the non-normalized data as well as the results obtained post-normalization to each of the normalizing genes/proteins chosen may enable more reliable comparison among studies.

Perhaps the use of normalizing genes in psychiatry research should be revised. The following alternatives may be considered: (1) Utilizing methodologies to obtain higher precision of sample concentration. (2) Running duplicate/triplicate measurements of each sample, dismissing results exhibiting extreme standard deviation among the replicates relative to the expression level. (3) Comparing the expression ratios between two genes responding in opposite directions (e.g. the anti-apoptotic *BCL2* and the pro-apoptotic *Bax*) rather than individual genes. Each of the above might improve our ability to reveal true biological effects.

## Methods

All procedures involving animals were reviewed and approved by the Ben-Gurion University animal experimentation ethics committee. The methods were carried out in accordance with the approved guidelines.

### Animals

Male, 10–12 weeks old wildtype mice from the *IMPA1* colony^68^ were used. Animals were maintained on a 12 h/12 h light/dark cycle (lights on between 8:00 a.m and 8:00 p.m.) with *ad libitum* access to food and water. Sample collection was performed during the light phase of the cycle between 9:00 am and 7:00 pm. All experiments were performed in accordance with the Ben-Gurion University animal experimentation ethics committee guidelines and regulations.

### Chronic lithium treatment

WT mice were divided into two groups (control and Li-treatment) and subjected to lithium-supplemented food or regular food for two weeks, as previously described^69^. At the end of the treatment, blood was extracted using cardiac puncture. Lithium plasma levels were measured in an ion-selective electrode apparatus ISE (AVL 9180 Electrolyte Analyzer, Hoffmann-La Roche, Basel, Switzerland). The measured lithium levels were in the range of 0.52–0.91 mM.

### RNA extraction

Total RNA was extracted from hippocampi specimens using the TRI reagent (Sigma-Aldrich, St. Louis, MO) followed by purification using the RNeasy kit (Qiagen, Germantown, MD). RNA concentration was determined spectrophotometrically (NanoDrop 2000, Thermo Fisher Scientific, Waltham, MA).

### Real-time PCR

RNA was reverse transcribed using Verso cDNA (Thermo Fisher Scientific, Waltham, MA). Real-time PCR was performed using ABsolute™ Blue Syber mix (ABgene, Lithuania) and Eco qPCR system (Illumina, San Diego, CA). The thermal cycler program was as follows: hold on 95 °C for 15 min, followed by 40 cycles of: 10 sec at 95 °C, 15 sec at 60 °C, 40 sec at 72 °C. The relative expression of each gene was calculated using the Pfaffl method^70^ implemented in the Eco qPCR system software. Samples were run in duplicates. Only samples with standard deviation (SD) < 0.05 between the duplicates were used in the analysis. Each qPCR plate contained equal number of samples from each of the groups. Samples with normalized expression values > |1.96| SD from the mean were removed from the analysis. Wilcoxon rank sum test was used to determine the significance of the results.

The expression of *BCL2 and MARCKS* was evaluated separately with each of the three normalizing genes *ACTB*, *MAPK6* or *ANKRD11*. We chose these genes for the following reasons: (1) *MAPK6* was recommended as a brain-reference gene^15^. Previous study from Padmos *et al*.^71^ reported no effect of lithium treatment on MAPK6 expression in human monocyte. Unpublished data from our microarrays study^16^ confirmed that brain *MAPK6* expression is not affected by lithium treatment (*SMIT1* colony: p > 0.23; *IMPA1* colony: p > 0.29, raw data is available upon request). (2) *ACTB* is the most commonly used normalizing gene in brain qPCR studies. (3) Based on the RefGenes tool of the Genevestigator software^60^ *ANKRD11* is the most stable gene in mouse hippocampus that was not affected by lithium treatment in our previous microarray study^16^ (*SMIT1* colony: p > 0.8; *IMPA1* colony: p > 0.14), as discussed in detail under the Results section. Supplementary Table S1 lists the primers’ sequences for the genes examined and the efficiencies of their reactions. The corresponding r^2^ values of the standard curves calculated for each of the genes were > 0.99.

### Estimation of gene expression stability

The RefGenes tool of the Genevestigator software platform was used according to the online tutorial (http://www.refgenes.org/rg/doc/ tutorial.jsp) in order to evaluate gene expression stability. Sample sets included in the analysis fulfilled the following criteria: Mouse 430_2: 40 k array/only WT genetic background/hippocampal samples. This filter resulted in a total of 80 arrays which were used to calculate the median and the standard deviation (SD) of *BCL2*, *ACTB*, *MAPK6* as well as *GAPDH*, another commonly used normalizing gene^72^. Calculation of the expected maximal fold of change relative to the mean expression signal for each of these genes was carried out using the inversed log_2_ of 1.96*SD^73^.

### Analysis of publicly available microarray datasets of the effect of lithium treatment

We performed differential expression analysis of two publicly available gene expression datasets – GSE66277 and GSE35291. GSE66277 contains expression profiles from three rat brain regions (cortex, hippocampus and striatum) following chronic treatment with different antipsychotics and mood-stabilizers (including lithium). GSE35291 contains expression profiles of hematopoietic progenitors following a one-week treatment with lithium or valproic acid. For GSE66277 signal intensities were quantile-normalized and log_2_-transformed using the RMA function from the R Bioconductor ‘affy’ package. For GSE35291 we used the log_2_ quantile-normalized intensities available on the Gene Expression Omnibus (GEO) site. We next mapped each probeset to a corresponding gene using Gemma annotation files^74^. Differential analyses were performed using the eBayes function from ‘limma’ R Bioconductor package. Benjamini–Hochberg procedure was used to control the false discovery rate (FDR).

## Electronic supplementary material

Supplementary data
