# Supplementary material for: The observed alteration in BCL2 expression following lithium treatment is influenced by the choice of normalization method
Source: Sci Rep. 2018 Apr 23;8:6399. doi: 10.1038/s41598-018-24546-1 (PMC5913222; doi:10.1038/s41598-018-24546-1)
Supplement: Supplementary file 1 — Supplementary data [file 41598_2018_24546_MOESM1_ESM.pdf]

Supplementary data

The observed alteration in BCL2 expression following lithium treatment is influenced by the choice of normalization method

Damri Odeya[1,2], Agam Galila[1,2*], Toker Lilah[3,4*]

[1]Department of Clinical Biochemistry and Pharmacology Ben-Gurion University of the Negev, Beer-Sheva, Israel; [2]Psychiatry Research Unit, Faculty of Health Sciences, Mental Health Center, Beer-Sheva, Israel; [3]Department of Psychiatry, University of British Columbia, BC, Canada; [4]Michael Smith Laboratories, University of British Columbia, BC, Canada

Table S1: **Primer sequences and reaction efficiencies**

	Sequence	Reaction efficiency
MAPK6	Fw: TATCGATGAGGTGCAGCTTG<br>Rev: GTTCTCGTGGTGATCTGGGT	94.5%
ACTB	Fw: TACTCTGTGTGGATCGGTG<br>Rev: GCTCAGTAACAGTCCGCCT	90.3%
ANKRD11	Fw: AAGGACTTTGCAGGCTGGAC<br>Rev: TGAGGATTTCCGCCATACCG	106.3
BCL2	Fw: GATGACTTCTCTCGTCGCTACC<br>Rev: CATCCCTGAAGAGTTCCTCCAC	100.4%
MARCKS	Fw: GTGGTATTGATGGAGCGGTG<br>Rev: TGCACACACTGCCAATACAG	96.7%
